# Predominance of *Acinetobacter* spp., Harboring the *bla*_IMP_ Gene, Contaminating the Hospital Environment in a Tertiary Hospital in Mwanza, Tanzania: A Cross-Sectional Laboratory-Based Study

**DOI:** 10.3390/pathogens11010063

**Published:** 2022-01-04

**Authors:** Vitus Silago, Eveline C. Mruma, Betrand Msemwa, Conjester I. Mtemisika, Shukurani Phillip, Reuben A. Ndagula, Maria M. Said, Martha F. Mushi, Stephen E. Mshana

**Affiliations:** 1 Department of Microbiology and Immunology, Weill Bugando School of Medicine, Catholic University of Health and Allied Sciences, Mwanza P.O. Box 1464, Tanzania; chrisslyne1@gmail.com (E.C.M.); msemwab700@gmail.com (B.M.); conjestermtemisika@yahoo.com (C.I.M.); phillipshukrani@yahoo.com (S.P.); mariahsaidy@gmail.com (M.M.S.); mushimartha@gmail.com (M.F.M.); stephen72mshana@gmail.com (S.E.M.); 2 Molecular Biology Department, Central Pathology Laboratory, Bugando Medical Centre, Mwanza P.O. Box 1370, Tanzania; 3 Institute of Allied Health Sciences, Ruaha Catholic University, Iringa P.O. Box 774, Tanzania; 4 National Public Health Laboratory, Dar es Salaam P.O. Box 9083, Tanzania; reubenabedneg@gmail.com; 5 Department of Clinical Laboratory, Kondoa District Hospital, Dodoma P.O. Box 40, Tanzania

**Keywords:** antimicrobial resistance, carbapenem resistance, carbapenemase genes, Gram-negative bacteria, modified Hodge test, double-disk synergy test, combination disk test, Mwanza

## Abstract

Data on colonization and hospital contamination of carbapenem-resistant Gram-negative bacteria (CR-GNB) are limited in low- and middle-income countries. We designed this study to determine the prevalence and co-existence of carbapenemase genes among CR-GNB isolated from clinical, colonization, and hospital environmental samples at a tertiary hospital in Mwanza, Tanzania. The modified Hodge test (MHT), the combined disk test (CDT), and the double-disk synergy test (DDST) were used for the phenotypic detection of carbapenemases. A multiplex PCR assay was used to detect *bla*_IMP_ and *bla*_KPC_, and a singleplex PCR assay was used to detect *bla*_OXA-48_. Data were analyzed by STATA version 13.0. Overall, 68.8% (44/64) of the CR-GNB had at least one phenotype by phenotypic methods, whereby 60.9% (39/64) were both CDT and DDST positive and 31.3% (20/64) were MHT positive. A total of 23/64 (35.9%) had at least one of the genes tested with the predominance of *bla*_IMP_ (91.3%; 21/23). In addition, 47.7% (21/44) of the CR-GNB phenotypes had at least one gene. Around 47.8% (11/23) of the CR-GNB carried multiple genes encoding for carbapenem resistance, with the maximum co-existence of *bla*_IMP_/*bla*_KPC_/*bla*_OXA-48_ (45.5%; 5/11). The majority of carbapenem-resistant genes were detected in *Acinetobacter* spp. (82.6%; 19/23) and isolated from bed swabs (69.6%; 16/23). *Acinetobacter* spp. carrying the *bla*_IMP_ gene predominantly contaminated the hospital environment. Therefore, we recommend routine decontamination of inanimate hospital surfaces, including patient beds.

## 1. Introduction

The upsurge in antimicrobial resistance (AMR) is associated with increased mortalities from unsuccessful antibiotic treatments [1]. Therefore, the World Health Organization (WHO) has declared that AMR is among the top 10 global public health crises facing humanity [1]. A high rate of antibiotic resistance is reported worldwide, indicating that we are running out of effective antibiotics [1]. However, the situation is worse in intensive care units (ICUs), where overuse of antibiotics is high. ICUs are defined as the epicenters of AMR, often described as an important determinant of patients’ outcomes [2,3]. Commonly, in ICUs, patients develop health-care-associated infections, namely central-line-associated bloodstream infections, ventilator-associated pneumonia, and catheter-associated urinary tract infections, from multidrug-resistant (MDR) bacteria, often acquired from contaminated hospital surfaces or invasive medical devices [4,5]. These bacterial pathogens successfully survive decontamination of hospital surfaces, whereby some bacteria, e.g., *Pseudomonas aeruginosa*, use biofilm formation and persist for days on hospital surfaces [4,6].

The most common MDR bacteria contaminating hospital surfaces or medical devices and causing health-care-associated infections in ICUs and other units are *Acinetobacter baumannii*, *Pseudomonas aeruginosa*, the *Klebsiella pneumoniae* complex, and *Escherichia coli* [4,5]. These bacteria are increasingly prevalent in causing health-care-associated MDR infections in ICUs and are resistant to multiple antibiotics, including carbapenems, which are considered the “last resort” and are reserved for managing MDR bacterial infections [4,7]. The emergence of carbapenem-resistant Gram-negative bacteria (CR-GNB) is associated with the increasing use of carbapenems in clinical settings, mainly in ICUs, limiting antibiotic options for treating MDR infections [8,9]. Therefore, prevention and control of health-care-associated infections from CR-GNB by identifying potential sources/reservoirs of CR-GNB is paramount.

To reduce antimicrobial resistance, in 2019, the WHO developed a tool to assist antibiotic stewardship at local, national, and global levels. To emphasize the importance of appropriate use of antibiotics, three groups, the Access, the Watch, and the Reserve (AWaRe), were developed. Carbapenems are classified into the Watch group, which includes antibiotics with high risk of selection for bacterial resistance and, therefore, should be prioritized for stewardship programs and monitoring.

It is well established that cross transmission of CR-GNB between patients and their immediate inanimate environment and translocation from rectal carriage play an important role during outbreaks of CR bacterial infections [10,11,12,13]. A study by Shimose et al. reported that about 15.5% of environmental samples in ICU rooms that were occupied by patients colonized with CR-*Acinetobacter baumannii* were also positive for CR-*Acinetobacter baumannii* [11]. Another study, by Lerner et al., reported that for 30 out of 34 patients who carried CR-Enterobacteriaceae (CRE), their surroundings, e.g., pillows (33%), infusion pumps (16%), and bedside tables (14%), were also contaminated with CRE at least once [12]. Aspelund et al. reported that during an outbreak of metallo-β-lactamase-producing *P. aeruginosa* (Pae-MBL), they identified 12 sinks in patients’ bathrooms that were contaminated with Pae-MBL exhibiting similar antibiotic susceptibility patterns and identical band patterns on pulse-field gel electrophoresis (PFGE) [13].

In Mwanza, Tanzania, Mushi et al. documented that 35.2% of MDR Gram-negative clinical isolates were carrying at least one carbapenemase gene whereby 61.3% carried the *bla*_IMP_ gene. Predominantly, carbapenemase genes were carried by the *K. pneumoniae* complex (11%) [14]. There is evidence of carriage of carbapenemase genes among MDR-GNB of clinical origin in this setting, although information on carriage of carbapenemase genes among CR-GNB of colonization and hospital environment origins is scarce. Therefore, we designed this study to determine the prevalence and co-existence of genes encoding for carbapenem resistance in GNB showing resistance to meropenem at the Bugando Medical Centre in Mwanza, Tanzania. 

## 2. Materials and Methods

### 2.1. Recovery of Bacterial Isolates

This cross-sectional laboratory-based study was conducted between June and August 2021. This study was carried out in Microbiology and Molecular Biology research laboratories of the Catholic University of Health and Allied Sciences (CUHAS) in Mwanza, Tanzania. 

Known GNB isolates archived at −80 °C with resistance to meropenem that were contemporaneously isolated from clinical (urine and blood), colonization (rectal swabs), and hospital environmental (patients’ bed swabs) samples from a previous study [15] were recovered for this study.

Isolates were recovered by subculturing on MacConkey agar (MCA; HiMedia, India) plates, which were incubated aerobically at 37 °C for 18–20 h. One to two colonies from culture plates were suspended in 5 mL of 0.85% sterile normal saline. Then, suspensions were adjusted to 0.5 McFarland standard (Densicheck; bioMérieux, Grassina, Italy) for phenotypic detection of carbapenemases production.

### 2.2. Phenotypic Detection of Carbapenemases Production

The modified Hodge test (MHT), the combined disk test (CDT), and the double-disk synergy test (DDST) were used for the phenotypic detection of carbapenemases production in CR-GNB, as described previously by Anwar et al. [16].

### 2.3. Molecular Characterization of Carbapenemase Genes

#### 2.3.1. DNA Extraction

DNA samples were extracted from test and control organisms grown on MCA using the QIAmp^®^ DNA Mini kit (QIAGEN, Hilden, Germany) following the manufacturer’s instructions and used for PCR detection of carbapenemases. The quantification, purity, and storage of the extracted DNA were carried out as described by Silago et al. 2021 [17].

#### 2.3.2. Multiplex PCR Technique for the Amplification of *bla*_IMP_ and *bla*_KPC_ Genes

A modified protocol of the multiplex PCR technique by Dallenne et al. 2010 [18] was used for the amplification of *bla*_IMP_ and *bla*_KPC_ genes. The *bla*_IMP_ gene (amplicon size 139 bp; New England BioLabs, Hertfordshire, UK) was amplified using the forward primer 5′-TTGACCACTCCATTTACDG-3′ and the reverse primer 5′-GATYGAGAATTAAGCCACYCT-3′, whereas the *bla*_KPC_ gene (amplicon size 538 bp; New England, BioLabs, UK) was amplified using the forward primer 5′-CATTCAAGGGCTTTCTTGCTGC-3′ and the reverse primer 5′-ACGACGGCATAGTCATTTGC-3′. Briefly, 4 µL of the DNA sample was subjected to each multiplex PCR in a 25 µL reaction mixture containing 2.5 µL of a PCR buffer (10×), 2 µL of Q-solution, 0.5 µL of dNTPs (10 nM), 1 µL of MgCl_2_ (50 mM), 0.3 µL of Taq polymerase, 3 µL of each primer (10 mM), and 2.7 µL of nuclease-free water. Amplification was carried out on a thermal cycler (Bio-Rad; Thermo-Fishers scientific, Jurong, Singapore). The initial step was activation (at 94 °C for 10 min), followed by 35 cycles of denaturation at 94 °C for 40 s, annealing at 55 °C for 40 s, and elongation at 72 °C for 60 s and the final elongation at 72 °C for 7 min. The PCR product were visualized under UV light using a gel documentation system (Vilber; Seoul, Korea) after running a gel electrophoresis at 100 V for 1 h on 1.5% agarose gel stained with SYBR DNA safe stain.

#### 2.3.3. Singleplex PCR Technique for the Amplification of the *bla*_OXA-48_ Gene

A similar protocol of the multiplex PCR technique described above was used for the singleplex PCR technique for the amplification of the *bla*_OXA-48_ gene at an annealing temperature of 57 °C. The *bla*_OXA-48_ gene (amplicon size 281 bp; New England, BioLabs, Hertfordshire, UK) was amplified using the forward primer 5′-GCTTGATCGCCCTCGATT-3′ and the reverse primer 5′-GATTTGCTCCGTGGCCGAAA-3′ by Dallenne et al. 2010 [18].

### 2.4. Quality Control

Known carbapenemases producing organisms characterized previously [14] and NCTC 13846 were used as control organisms.

### 2.5. Data Management and Analysis

Data were entered into MS Excel for cleaning and coding and analyzed by STATA 13.0 computer software. Percentages and fractions were used to summarize categorical data. Univariate logistic regression analysis was used for testing phenotypic methods to predict carriage of genes encoding for carbapenem resistance. A *p*-value of less than 0.05 at a 95% confidence interval (95% CI) was considered statistically significant.

### 2.6. Ethical Considerations

The protocols used in this study were reviewed and approved by the joint Catholic University of Health and Allied Sciences (CUHAS) and the Bugando Medical Centre (BMC) Research Ethics and Review Board. Ethical clearance certificate number CREC: 1890/2021 was provided.

## 3. Results

### 3.1. Sources and Species of Isolates Used in This Study

A total of 64 CR-GNB were recovered during this study, of which the majority were recovered from ICUs (62.5%; 40/64) and bed swabs (56.3%; 36/64) and were *Acinetobacter* spp. (67.2%; 43.64) (Table 1).

### 3.2. Phenotypic Detection and Molecular Characterization of Carbapenemase Genes

**Figure 1:**
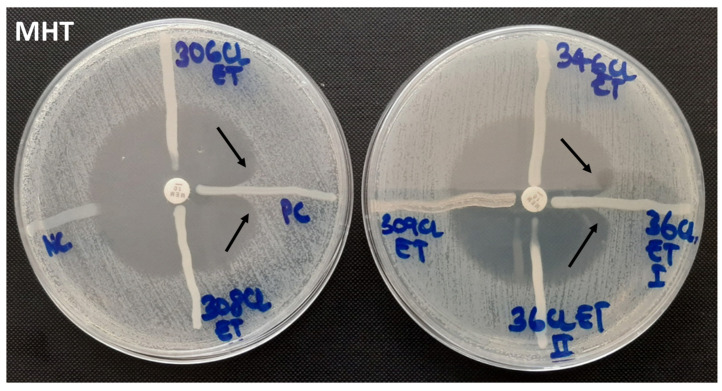
Modified Hodge test showing negative and positive controls (**left**) and negative and positive test organisms (**right**).

**Figure 2:**
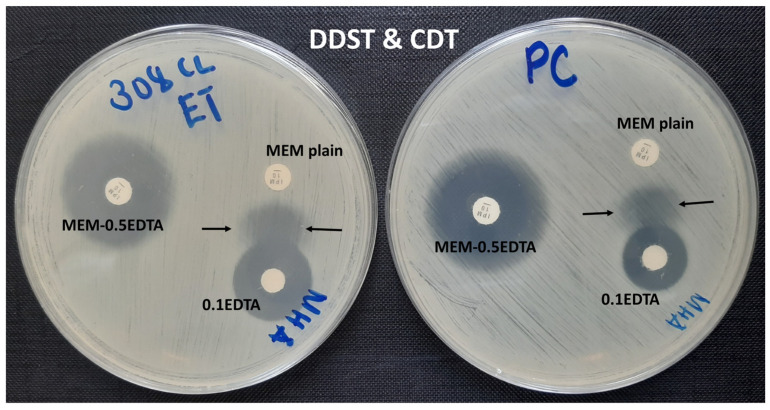
Combine disk test (CDT; MEM plain and MEM-0.5EDTA) and double-disk synergy test (DDST; MEM plain and 0.1EDTA) performed on the same plate, showing a positive result for the test organism (**left**) and a positive result for the control organism (**right**).

**Figure 3:**
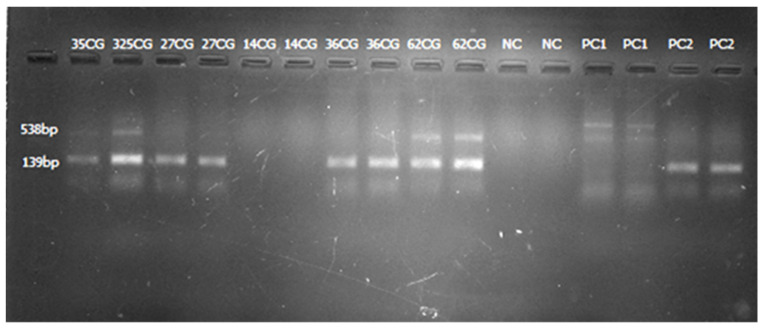
Gel image from multiplex PCR amplification showing positive *bla*_IMP_ (lanes 2 and 3, 4 and 5, 8 and 9, and 10 and 11; samples run in duplicate), positive *bla*_KPC_ (lanes 2 and 3; lanes 10 and 11; samples run in duplicate), and positive controls for *bla*_KPC_ (lanes 14 and 15) and *bla*_IMP_ (lanes 16 and 17).

The overall carbapenem resistance phenotypes were observed in 68.8% (44/64) of the samples, of which 31.3% (20/64) were observed by MHT and 60.9% (39/64) by CDT and DDST (both) (Figure 1 and Figure 2). The three phenotypes-based methods, MHT, CDT, and DDST, correlated in 23.4% (15/64) of the CR-GNB tested. About 35.9% (23/64) of the GNB resistant to meropenem carried at least one gene encoding for carbapenem resistance (Figure 3). The predominant gene detected was *bla*_IMP_ (91.3%; 21/23). The co-existence of genes encoding for carbapenem resistance was detected in 47.8% (11/23) of the samples, and *bla*_IMP_/*bla*_KPC_/*bla*_OXA-48_ (45.5%; 5/11) was the most observed combination (Table 2). Further, about 45.5% (20/44) of the CR-GNB with at least one phenotype by either MHT (20%; 4/20) or CDT and DDST (80%; 16/20) carried no gene encoding for carbapenem resistance tested in our study. However, about 4.3% (1/23) of the GNB carrying genes encoding for carbapenem resistance, *bla*_IMP_ and *bla*_KPC_, had negative phenotypes by all phenotype-based methods used in this study.

### 3.3. The Distribution of Genes Encoding for Carbapenem Resistance by Unit, Origin, and Isolate

The majority of the CR-GNB carrying at least one gene encoding for carbapenem resistance were isolated from the neonatology unit (60.9%; 14/23) and from bed swabs (69.6%; 16/23). Moreover, *Acinetobacter* spp. were frequently detected (82.6%; 19/23) carrying genes encoding for carbapenem resistance (Table 3).

### 3.4. Phenotypic Methods Predict Carriage of Genes Encoding for Carbapenem Resistance

By univariate logistic regression analysis, CDT (OR: 12.0; 95% CI: 2.48–58.05; *p* = 0.002), DDST (OR: 10.9; 95% CI: 2.26–52.79; *p* = 0.003), and MHT (OR: 4.2; 95% CI: 1.34–12.84; *p* = 0.013) significantly predicted carriage of the *bla*_IMP_ gene. MHT (OR: 14.0; 95% CI: 2.62–74.89; *p* = 0.002) significantly predicted carriage of the *bla*_KPC_ gene. For the *bla*_OXA-48_ gene, all three phenotypic methods (CDT, DDST, and MHT) had a collinearity result and, therefore, it was difficult to determine the level of statistical significance to predict carriage of the *bla*_OXA-48_ gene (Table 4).

## 4. Discussion

Sixty-four GNB with resistance to meropenem contemporaneously isolated from different sources (rectal and bed swabs and blood and urine samples) were recovered during this study for phenotypic detection and molecular characterization of genes encoding for carbapenem resistance. Most of the GNB with resistance to meropenem were isolated from the premature unit and the AICU, from bed swab samples, and the predominant isolate was *Acinetobacter* spp. Generally, *Acinetobacter* spp. is well equipped with numerous intrinsic and acquired mechanisms of antimicrobial resistance [19]. Moreover, *Acinetobacter* spp. are the commonest bacteria contaminating hospitals’ surfaces, especially in ICUs and highly dependent units (HDUs; i.e., premature units), where antimicrobial pressure is high. Therefore, the majority of the CR-GNB recovered during this study were *Acinetobacter* spp. isolated from ICUs and the premature unit and mostly contaminating patients’ beds. A high proportion of MDR bacterial contamination in ICUs and HDUs is significantly contributed by the overuse of antibiotics, which is associated with the emergence of MDR bacteria and overcrowding of patients, which facilitates their transmission and contamination of inanimate hospital environments.

To date, different phenotypic methods are in use for the detection of carbapenem resistance globally [16,20]. MHT, CDT, and DDST are among the common methods used for phenotypic detection and typing of carbapenem resistance, i.e., CDT and DDST are good predictors of metallo-β-lactamases (e.g., *bla*_IMP_, *bla*_VIM_, and *bla*_NDM_) while MHT is a good predictor of non-metallo-β-lactamases (e.g., *bla*_KPC_ and *bla*_OXA-48_) [21,22,23]. In the current study, MHT, CDT, and DDST significantly predicted carriage of the *bla*_IMP_ gene, whereas MHT significantly predicted carriage of the *bla*_KPC_ gene in CR-GNB. About one-third and nearly two-thirds of the GNB showing resistance to meropenem had positive phenotypes by MHT and CDT or DDST, respectively. Similar findings were reported elsewhere, that MHT has low sensitivity and specificity rates, failing to detect MBLs producing GNB [24] and also that the high proportion of MBLs detection by CDT and DDST may be due to the good dissemination and predominance of genes encoding for MBLs locally and globally [14,25,26,27].

In the current study, about one-third of the CR-GNB had at least one gene encoding for carbapenem resistance, with the predominance of the *bla*_IMP_ gene. Mushi et al. reported similar findings in 2014 from the same setting, although they used clinical isolates [14], while a large proportion of isolates in the current study were recovered from hospital environments, i.e., patients’ beds (56.3%; 36/64). In our study, the predominant CR-GNB carrying carbapenemase genes was *bla*_IMP_, in *Acinetobacter* spp., contaminating hospital environments, i.e., patients’ beds (neonatal cots) in the premature unit. Similar to previous studies, [28,29,30], this study provides evidence that patients’ immediate environments in ICUs and HDUs act as reservoirs of CR-GNB and may play the primary role in the spreading of CR-GNB pathogens, leading to the emergence of multidrug-resistant infections in these units. Moreover, co-existence of genes encoding for carbapenem resistance was observed in nearly one-half (47.8%) of CR-GNB harboring genes encoding for carbapenem resistance, of which 45.5% had a co-existence of three (*bla*_IMP_/*bla*_KPC_/*bla*_OXA-48_) genes. Our findings, co-existence of carbapenemase encoding genes in CR-GNB, are in line with other studies in the same setting [14] and elsewhere [24]. Genes encoding for carbapenem resistance are commonly harbored in conjugative plasmids that harbor multiple-antibiotic-resistant genes (ARGs) [31]. In addition, some bacteria carry multiple plasmids that harbor different carbapenemase genes [32].

We also observed that 4 out of 20 CR-GNB with carbapenemase phenotypes by MHT and 16 out of 20 with carbapenemase phenotypes by CDT/DDST had no gene encoding for carbapenem resistance out of those covered in this study. False positive results by MTH have been reported elsewhere [33,34]. First, a low level of carbapenem hydrolysis by extended spectrum β-lactamases (ESBLs), particularly the CTX-M types, has been presumed to be a reason for false positive MHT [33,34]. Second, these four isolates with false positive MHT phenotypes may be producing other β-lactamases, such as Amp-*C* β-lactamase, and other genes encoding for MBLs (e.g., *bla*_VIM_ and *bla*_NMD_), which were not covered in our study. Studies are reporting that adding cloxacillin and zinc sulfate or boronic acid to MHA plates when performing MHT prevents false positive results from other β-lactamase production [16,35,36]. Unfortunately, in the current study, we did not supplement the MHA plates with either cloxacillin or zinc sulfate/boronic acid. Sixteen isolates with positive phenotypes by CDT and DDST but neither gene may be harboring other MBLs (e.g., *bla*_VIM_ and *bla*_NMD_), which were not covered in the current study. The presence of *bla*_VIM_ and *bla*_NMD_ genes was reported among clinical isolates from the same setting [14]. Therefore, these 16 isolates with positive phenotypes by CDT and DDST may be harboring these MBLs. However, 1 out of 23 CR-GNB carrying genes encoding for carbapenem resistance was negative by both phenotype-based methods; MHT and CDT and DDST. The isolate was *Acinetobacter* spp., carrying two genes, *bla*_IMP_ and *bla*_KPC_. This may be due to pseudo-genes, which are nonfunctional genes resembling functional genes but have undergone one or more mutations eliminating their ability to be expressed and detected phenotypically, a phenomenon documented previously [37,38].

## 5. Conclusions

In this study, we observed that about one-third of the CR-GNB, predominantly *Acinetobacter* spp., commonly contaminating patients’ beds in ICUs and the premature unit were carrying at least one gene encoding for carbapenem resistance. The predominant gene and the common co-existence genes detected were *bla*_IMP_ and *bla*_IMP_/*bla*_KPC_/*bla*_OXA-48_, respectively. We, therefore, recommend the implementation of infection prevention and control measures, particularly hand hygiene and hospital environmental cleaning.

## Figures and Tables

**Table 1:** Sources and species of isolates used in this study.

Variables		Frequency (*n*)	Percentages (%)
Ward	Neonatal ICU (NICU)	10	15.6
	Premature unit	24	37.5
	Adult ICU (AICU)	30	46.8
Source	Blood	9	14.1
	Urine	5	7.8
	Rectal swab	14	21.8
	Bed swab	36	56.3
Isolate name	*K. pneumoniae* complex	10	15.6
	*Acinetobacter* spp.	43	67.2
	*E. coli*	7	10.9
	*P. aeruginosa*	2	3.1
	Unidentified GNRs	2	3.1
Bed swab (*N* = 36)	*Acinetobacter* spp.	28	77.8
	*K. pneumoniae* complex	4	11.1
	*P. aeruginosa*	2	5.6
	Unidentified GNRs	2	5.6
Rectal swab (*N* = 14)	*Acinetobacter* spp.	8	57.1
	*K. pneumoniae* complex	2	14.3
	*E. coli*	4	28.6
Blood (*N* = 9)	*Acinetobacter* spp.	5	55.6
	*K. pneumoniae* complex	4	44.4
Urine (*N* = 5)	*E. coli*	3	60.0
	*Acinetobacter* spp.	2	40.0

Key: ICU = intensive care unit; GNRs = Gram-negative rods.

**Table 2:** Phenotypic detection and molecular characterization of carbapenemase genes.

Variables		Frequency (*n*)	Percentages (%)
Modified Hodge test (*N* = 64)	Negative	44	68.7
	Positive	20	31.3
Combined disk test (*N* = 64)	Negative	26	39.1
	Positive	39	60.9
Double-disk synergy test (*N* = 64)	Negative	25	39.1
	Positive	39	60.9
Carrying at least one carbapenemase gene (*N* = 64)	No	41	64.1
	Yes	23	35.9
Proportions of carbapenemase genes carried (*N* = 23)	*bla*_IMP_	21	91.3
	*bla*_KPC_	10	43.5
	*bla*_OXA-48_	8	34.8
Carriage of more than one carbapenemase gene (*N* = 23)	No	12	52.2
	Yes	11	47.8
Combinations of carbapenemase genes (*N* = 11)	*bla*_IMP_/*bla*_KPC_/*bla*_OXA-48_	5	45.5
	*bla*_IMP_/*bla*_KPC_	4	36.4
	*bla*_IMP_ */bla*_OXA-48_	1	4.3
	*bla*_KPC_ */bla*_OXA-48_	1	4.3

**Table 3:** The distribution of genes encoding for carbapenem resistance.

Variables		Frequency (*n*)	Percentage (%)
Unit	NICU	3	13.0
	Premature unit	14	60.9
	AICU	6	26.9
Source	Blood	4	17.4
	Urine	0	0.0
	Rectal swab	2	8.7
	Bed swab	17	73.9
CR-GNB	*K. pneumoniae* complex	0	0.0
	*Acinetobacter* spp.	19	82.6
	*E. coli*	1	4.3
	*P. aeruginosa*	1	4.3
	Unidentified GNR	2	8.7
Bed (*N* = 16)	*Acinetobacter* spp.	13	81.3
	Unidentified GNRs	2	12.5
	*P. aeruginosa*	1	6.2
Rectal swab (*N* = 3)	*Acinetobacter* spp.	2	66.7
	*E. coli*	1	33.3
Blood (*N* = 4)	*Acinetobacter* spp.	4	100

**Table 4:** Phenotypic methods predict carriage of genes encoding for carbapenem resistance.

Variables		Prediction of *bla*_IMP_ Gene				Prediction of *bla*_KPC_ Gene			
		POS *n* (%)	NEG *n* (%)	OR (95% CI)	*p*-Value	POS *n* (%)	NEG *n* (%)	OR (95% CI)	*p*-Value
CDT	Positive	19 (50.0)	24 (92.3)	12 (2.48–58.05)	0.002	9 (23.7)	29 (76.3)	7.76 (0.92–65.5)	0.060
	Negative	2 (7.7)	19 (50.0)			1 (3.8)	25 (96.2)		
DDST	Positive	19 (32.8)	20 (51.3)	10.93 (2.26–52.79)	0.003	9 (23.1)	30 (76.9)	7.2 (0.85–60.86)	0.070
	Negative	2 (8.0)	23 (92.0)			1 (4.0)	24 (96.0)		
MHT	Positive	11 (55.0)	9 (45.0)	4.16 (1.34–12.84)	0.013	8 (40.0)	12 (60.0)	14 (2.62–74.89)	0.002
	Negative	10 (22.7)	34 (77.3)			2 (4.6)	42 (95.5)		

Key: CDT = combined disk test; CI = confidence interval; DDST = double disk synergy test; MHT = modified Hodge test; NEG = negative; OR = odd ratio; and POS = positive.

## Data Availability

The data presented in this study are available on request from the corresponding author.
